# Design‐ and model‐based recommendations for detecting and quantifying an amphibian pathogen in environmental samples

**DOI:** 10.1002/ece3.3616

**Published:** 2017-11-12

**Authors:** Brittany A. Mosher, Kathryn P. Huyvaert, Tara Chestnut, Jacob L. Kerby, Joseph D. Madison, Larissa L. Bailey

**Affiliations:** ^1^ Department of Fish, Wildlife, and Conservation Biology Colorado State University Fort Collins CO USA; ^2^ US Geological Survey Oregon Water Science Center Portland OR USA; ^3^ Department of Biology University of South Dakota Vermillion SD USA

**Keywords:** *Batrachochytrium dendrobatidis*, Chytridiomycosis, detection probability, eDNA, filtration, host‐pathogen dynamics, qPCR, monitoring, multiscale occupancy

## Abstract

Accurate pathogen detection is essential for developing management strategies to address emerging infectious diseases, an increasingly prominent threat to wildlife. Sampling for free‐living pathogens outside of their hosts has benefits for inference and study efficiency, but is still uncommon. We used a laboratory experiment to evaluate the influences of pathogen concentration, water type, and qPCR inhibitors on the detection and quantification of *Batrachochytrium dendrobatidis* (*Bd*) using water filtration. We compared results pre‐ and post‐inhibitor removal, and assessed inferential differences when single versus multiple samples were collected across space or time. We found that qPCR inhibition influenced both *Bd* detection and quantification in natural water samples, resulting in biased inferences about *Bd* occurrence and abundance. Biases in occurrence could be mitigated by collecting multiple samples in space or time, but biases in *Bd* quantification were persistent. Differences in *Bd* concentration resulted in variation in detection probability, indicating that occupancy modeling could be used to explore factors influencing heterogeneity in *Bd* abundance among samples, sites, or over time. Our work will influence the design of studies involving amphibian disease dynamics and studies utilizing environmental DNA (eDNA) to understand species distributions.

## 1 INTRODUCTION

Emerging infectious diseases (EIDs) are a prominent threat to wildlife (Langwig et al., 2015) and are important drivers of local extinctions (Smith, Sax, & Lafferty, 2006). In addition to affecting host species, disease‐related declines can have cascading effects on community structure and ecosystem‐level processes (Hollings, Jones, Mooney, & McCallum, 2014; Jachowski et al., 2014; Whiles et al., 2013). Emerging infectious diseases in amphibian populations are on the rise, with ranavirus infections, saprolegniosis, *Ribeiroia* spp. infections, and chytridiomycosis contributing to mortality events (Daszak, Cunningham, & Hyatt, 2003). Chytridiomycosis is caused by the fungal pathogens *Batrachochytrium dendrobatidis* (*Bd*) and *Batrachochytrium salamandrivorans* (*Bsal*). *Bd* is implicated in the declines of over 200 anuran species across the globe (Skerratt et al., 2007), and, although *Bsal* is a newly identified pathogen causing disease in urodelans, it has already been linked to fire salamander (*Salamandra salamandra*) extirpations in the Netherlands (Martel et al., 2013). Both pathogens are of concern to natural resource scientists and managers, and key uncertainties about pathogen transmission, distributions, and dynamics within amphibian host populations remain (Grant et al., 2016; Venesky, Raffel, McMahon, & Rohr, 2014).

Pathogen detection is central to understanding host‐pathogen dynamics and to making informed management decisions (Voyles et al., 2014). Swabbing amphibian skin is the recommended (Hyatt et al., 2007) and most common method for detecting *Bd* and *Bsal*. Collecting skin swabs can be difficult at sites where host amphibian species are rare or extinct, but understanding the persistence and distributions of these pathogens in the environment in these places remains important and would provide valuable ecological and conservation insights. For instance, sites without amphibians are preferred for amphibian reintroduction initiatives (Muths, Bailey, & Watry, 2014) and *Bd* status must be assessed to maximize the probability of success of costly reintroductions. Relying on swabs can also make it difficult to answer basic ecological questions about pathogen persistence in the absence of amphibian hosts (Mosher, Bailey, Hubbard, & Huyvaert, 2017) or to assess the spatial or temporal distribution of *Bd* or *Bsal* in water bodies.

Water filtration can be used to detect *Bd*'s infective stage (Berger, Hyatt, Speare, & Longcore, 2005) without relying on amphibian host presence and detection. Filtration has been used to detect zoospores in rainwater (Kolby et al., 2015), from amphibians soaked in baths of distilled water (Hyatt et al., 2007; Shin, Bataille, Kosch, & Waldman, 2014), and in aquatic habitats such as amphibian breeding ponds (Chestnut et al., 2014; Schmidt, Kéry, Ursenbacher, Hyman, & Collins, 2013), making filtration an important technology for both aquatic and terrestrial amphibian communities. Filtration can be used to survey potential amphibian reintroduction sites currently devoid of hosts, yielding information about pathogen distributions independent of host distributions. Additionally, filtration could make survey efforts more efficient by eliminating capture and handling of amphibians and by allowing multiple independent sample types (e.g., visual encounter surveys, amphibian swabs, and *Bd* filtration samples) to be collected during a single site visit. The relationship between *Bd* detection and *Bd* concentration is largely unknown because the filtration method has not been experimentally assessed at low concentrations or abundances of *Bd* that are likely characteristic of natural settings. For filtration to become a useful field method, its utility for both detecting and quantifying pathogen DNA must be assessed.

Many modern molecular methods (e.g., quantitative real‐time polymerase chain reaction or qPCR) provide information about the occurrence and quantity of target DNA found in a sample. Quantities estimated from qPCR could be used to understand the relationship between infection load and disease risk for resident or reintroduced amphibians, but the validity of this index is not well‐supported for *Bd* swabs (Clare, Daniel, Garner, & Fisher, 2016) and has never been assessed for filtered water samples. Despite this lack of validation, quantitative estimates from qPCR have been used as both indices and true measures of *Bd* abundance (Miller, Talley, Lips, & Campbell Grant, 2012; Venesky, Liu, Sauer, & Rohr, 2014). Understanding the relationship between the estimated quantity of *Bd* and true *Bd* concentration is central to understanding infection thresholds (Vredenburg, Knapp, Tunstall, & Briggs, 2010), assessing impacts of management actions (Scheele et al. 2014), and targeting areas for reintroduction of declining amphibian species (Muths et al., 2014).

The presence of inhibitory agents (e.g., humic acid) in field samples can interfere with qPCR and cause errors (i.e., false negatives) which can bias biological inference. qPCR inhibition has been identified in amphibian swab samples (Kosch & Summers, 2013) and in filtered water samples where shed DNA is captured (McKee, Spear, & Pierson, 2015). The presence of qPCR inhibitors likely influences both the detection and quantification of *Bd* DNA, but the extent of this influence has not been explored.

We designed an experiment to evaluate the effects of *Bd* concentration, water type (distilled and natural), and qPCR inhibition on the detection and quantification of *Bd* captured using water filtration. We evaluated samples independently (single sample scenario) or in groups (multiple samples scenario) to mimic spatial and temporal replication in field studies. We chose concentrations of *Bd* that were low but biologically relevant to amphibians, as these concentrations will be most informative to those designing field studies, understanding disease dynamics, and developing conservation strategies. We assessed qPCR inhibition by comparing *Bd* detection and quantification in two water types (distilled and natural) and by analyzing samples with and without removing contaminants that can inhibit qPCR reactions. We discuss the implications of our work in the context of host‐pathogen ecology, study design, and ecological modeling and provide information that will be useful to researchers and managers seeking to better understand and conserve amphibian communities.

## 2 MATERIALS AND METHODS

### 2.1 Experimental and molecular methodology

**Figure 1:**
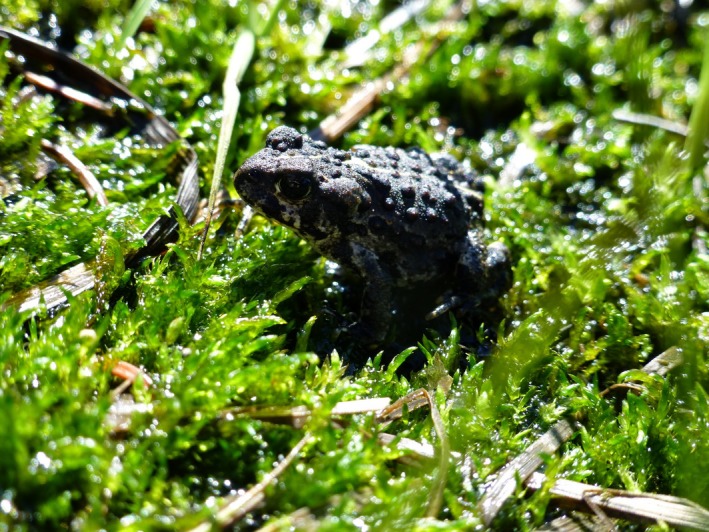
The boreal toad (*Anaxyrus boreas boreas*) is a high‐elevation amphibian that is in decline throughout the Southern Rocky Mountains, in part due to the fungal pathogen *Batrachochytrium dendrobatidis* and the disease chytridiomycosis. Photograph by Brittany A. Mosher

We cultured *Bd* strain JEL274, originally collected from a boreal toad (*Anaxyrus boreas boreas*; Figure 1) in Colorado, until zoospores were mature (Kirshtein, Anderson, Wood, Longcore, & Voytek, 2007). We used a hemocytometer and bright‐field microscopy to count live zoospores and to determine the concentration of the harvested cultures. We then diluted the culture solution with sterile deionized water to create solutions varying in *Bd* concentration.

We randomly assigned levels of two factors (concentration and water type) to 300 study units (250‐ml glass jars) and investigated *Bd* detection via water filtration at 5 concentrations: 0, 0.05, 0.175, 1, and 50 zoospores/ml. The 0 zoospore/ml group served as a negative control, while the 0.05 zoospore/ml group was included to explore the lower limit of detection of *Bd* (Boyle, Boyle, Olsen, Morgan, & Hyatt, 2004; Kerby, Schieffer, Brown, & Whitfield, 2012; Kirshtein et al., 2007). The highest concentration (50 zoospores/ml) was selected for its lethality to young‐of‐the‐year boreal toadlets experimentally bathed in this concentration for 72 hr, whereas the intermediate levels reflect concentrations that were sublethal to boreal toadlets and that likely exist in natural settings (Carey et al., 2006).

We considered two water types: distilled water and water from a natural, lotic source where *Bd* had never been detected. Natural water was selected to investigate how inhibitors influence pathogen detection, while distilled water was selected because it is commonly used to bathe amphibians prior to filtration (Shin et al., 2014) and in studies of molecular methodology (Bletz, Rebollar, & Harris, 2015). The natural water was autoclaved and allowed to sit for 20 days until the experiment to kill any *Bd* DNA and render it undetectable (Piotrowski, Annis, & Longcore, 2004; Thomsen et al., 2012). We included an equal number of jars (*n* = 36) for all groups except the control groups (*n* = 6).

We inoculated jars with known concentrations of *Bd* and let the jars rest for 18 hr prior to sampling. Upon sampling, we briefly agitated each jar and then drew one, 60‐ml sample from a total volume of 200 ml using a 0.22‐μm Sterivex capsule filter with a male Luer‐Lok (Millipore, Billerica, MA, USA) connected to a sterile 60‐ml syringe. After collection, we prepared the sample using lysis buffer according to the protocols of Chestnut et al. (2014). Samples were maintained at room temperature until DNA was extracted (within 17 weeks of collection).

We extracted DNA using Gentra Puregene Tissue Kits (Qiagen, Valencia, CA, USA; Chestnut et al., 2014). We initially analyzed the extracts in triplicate wells using the qPCR assay outlined by Boyle et al., (2004) and updated by Kerby et al. (2012), but we found low *Bd* detection probabilities in the natural water that provided evidence of PCR inhibition (see 3). Subsequently, we used a post‐extraction spin column purification kit (OneStep™ PCR Inhibitor Removal Kit; Zymo Research, Irvine, CA, USA) on each sample and analyzed the resulting post‐purification sample in triplicate with qPCR. Extraction and qPCR were conducted in separate laboratory spaces using dedicated supplies and workspaces. Aerosol‐barrier pipette tips were used, and all laboratory equipment and benches were cleaned with bleach in between procedures.

The target region for amplification during qPCR was the internal transcribed spacer region one (ITS1), which is variable in copy number per zoospore among *Bd* strains (Longo et al., 2013). We estimated the number of ITS1 copies present in single JEL274 zoospore by comparing the amplification rates between a known quantity of JEL274 zoospores and a qPCR standard made from PCR amplicons of the ITS1 region. We used this information to convert estimated number of copies from qPCR to estimated zoospore counts and used these counts to quantify bias in estimates of *Bd* concentration. We investigated the correlation between qPCR copy number and *Bd* concentration because the *Bd* strain is unknown in most field studies.

### 2.2 *Bd* occurrence and detection

**Figure 2:**
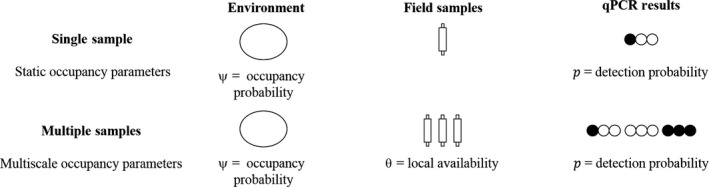
Single and multiple sample scenarios in an example field study of *Bd* occurrence in the aquatic environment. Single samples can be used to make inferences about *Bd* detection probability (*p*) and site‐level occupancy (*ψ*). If multiple samples are collected, additional inferences can be made about heterogeneity in *Bd* occurrence across space or time (θ). Resulting replicate qPCR results from both sampling strategies can be analyzed in an occupancy framework that accounts for imperfect detection

We investigated how two different sampling scenarios, single sample and multiple samples, might influence bias in estimates of *Bd* occurrence. In the single sample scenario, single jars were used as sample units. This scenario corresponds to collecting a single water filter (i.e., field sample) at a wetland (Figure 2). For the multiple samples scenario, we redefined a study unit as a collection of three jars (i.e., field samples) within the same treatment group to emulate field protocols where multiple filters are collected at a single site over space or time (e.g., Chestnut et al., 2014; Pilliod, Goldberg, Arkle, & Waits, 2014; Figure 2). This reduced our sample size from 36 to 12 study units per treatment, but increased the number of opportunities for detection within each study unit.

Occupancy models use repeated surveys to estimate the probability that a study unit is occupied by the species of interest, while explicitly allowing for imperfect species detection. We used results from all 3 qPCR replicates (hereafter, “wells”) per filter sample as repeat surveys to detect *Bd*. We modeled both *Bd* occurrence (*ψ*) and detection probability (*p*) as additive and interactive functions of *Bd* concentration and water type using standard occupancy models (MacKenzie et al., 2002) for the single sample scenario. For the multiple samples scenario, we used a multiscale occupancy model (Nichols et al., 2008) to accommodate multiple filter samples per study unit and multiple qPCR wells per sample. In this case, we estimated the probability that a study unit (group of three jars) was occupied by *Bd* (*ψ*), the probability that *Bd* was present in an individual filter sample given that the study unit was occupied (θ), and the probability of detecting *Bd* given that it was present on a filter (*p*). We modeled *ψ*, θ, and *p* as additive and interactive functions of *Bd* concentration and water type. In both sampling scenarios, we conditioned on samples that were inoculated with *Bd* and compared the estimated occupancy probabilities with the true values (*ψ*_*true*_ = θ_*true*_ = 1) to quantify estimation biases using the observed data. We evaluated competing models where concentration was treated as a continuous variable or a factor variable, but never allowed both variable types to appear in a single model. Models were fit to both pre‐ and post‐purification data.

### 2.3 Zoospore quantification

We assessed the validity of copy number as an index of *Bd* concentration in the single and multiple sample scenarios by calculating the Spearman's rank‐order correlation coefficient (*r*_s_) between the mean qPCR copy number for a sample and the known concentration. This nonparametric test was selected because we did not want to assume a strictly linear relationship between the mean qPCR copy number and the known concentrations, largely due to the extreme variation in magnitude of the experimental concentrations. We included nondetections (i.e., wells with qPCR copy number estimates of 0) and compared the correlation coefficients for both pre‐ and post‐purification datasets to assess the impact of qPCR inhibition on quantity estimation.

Next, we converted qPCR copy number to zoospore concentration using strain‐specific *Bd* information and used linear regression to evaluate if relative bias in the estimated concentration relative bias=(Estimated[Bd]−Experimental[Bd])/(Experimental[Bd]) was related to water type or known *Bd* concentration. Positive relative bias values indicate an overestimation of concentration via qPCR, while negative values indicate underestimation. We used relative bias as the response variable for this regression analysis because we expected that bias and variance would vary substantially among concentrations.

### 2.4 Software and multimodel inference

We used an information‐theoretic approach to rank candidate models using Akaike's Information Criterion corrected for small sample sizes (AICc; Burnham & Anderson, 2002). Occupancy models were fit using the R package “RMark” (Laake, 2013; White & Burnham, 1999). To account for model selection uncertainty, we report model‐averaged parameter estimates that consider all models. Spearman's rank‐order correlation and linear regression models for relative bias were conducted in R (R Development Core Team 2012).

## 3 RESULTS

Thirty‐three percent (4/12) of negative control jars yielded false‐positive results when purification was not performed, compared to 8% post‐purification (1/12 jars). The negative control jar that tested positive when inhibitors were removed did not test positive without removing inhibitors. We eliminated questionable detections in both datasets by imposing a threshold based on the highest copy number estimated in the negative controls (samples with fewer than 8.9 qPCR copies pre‐purification and 63.7 copies post‐purification were excluded). These samples were estimated to contain less than 1 zoospore.

The proportion of jars where *Bd* was detected in at least one qPCR well varied among concentrations and water types (Table 1). We detected *Bd* in at least one of three wells in 127 of 288 (44%) inoculated jars pre‐purification, and in at least one of three wells in 185 of 288 (64%) inoculated jars post‐purification.

**Table 1:** Estimated proportion of sample units that were occupied by *Batrachochytrium dendrobatidis* (*Bd*) using raw data and occupancy modeling approaches with both pre‐ and post‐purification datasets for four *Bd* concentrations (zoospores/ml)

*Bd*	Raw data				Single sample occupancy				Multiple samples occupancy			
	Pre‐purification		Post‐purification		Pre‐purification		Post‐purification		Pre‐purification		Post‐purification	
	Distilled	Natural	Distilled	Natural	Distilled	Natural	Distilled	Natural	Distilled	Natural	Distilled	Natural
0.05	0.47	0.11	0.06	0.56	0.72 (0.17)	0.16 (0.07)	0.10 (0.04)	0.54 (0.08)	1.00 (0.00)	1.00 (0.00)	1.00 (0.00)	1.00 (0.00)
0.175	0.53	0.25	0.31	0.81	0.66 (0.12)	0.23 (0.07)	0.31 (0.07)	0.82 (0.05)	1.00 (0.00)	1.00 (0.00)	1.00 (0.00)	1.00 (0.00)
1	0.69	0.28	0.58	0.95	0.73 (0.08)	0.27 (0.07)	0.60 (0.08)	0.94 (0.03)	1.00 (0.00)	1.00 (0.00)	1.00 (0.00)	1.00 (0.00)
50	0.94	0.25	0.92	0.97	0.86 (0.08)	0.31 (0.10)	0.90 (0.05)	0.99 (0.01)	1.00 (0.00)	1.00 (0.00)	1.00 (0.00)	1.00 (0.00)

Using raw detection data, a sample was classified as occupied if *Bd* was detected in at least one of the three qPCR wells. Model‐averaged estimates and unconditional standard errors (in parentheses) are given for occupancy approaches.

### 3.1 *Bd* occurrence and detection

**Figure 3:**
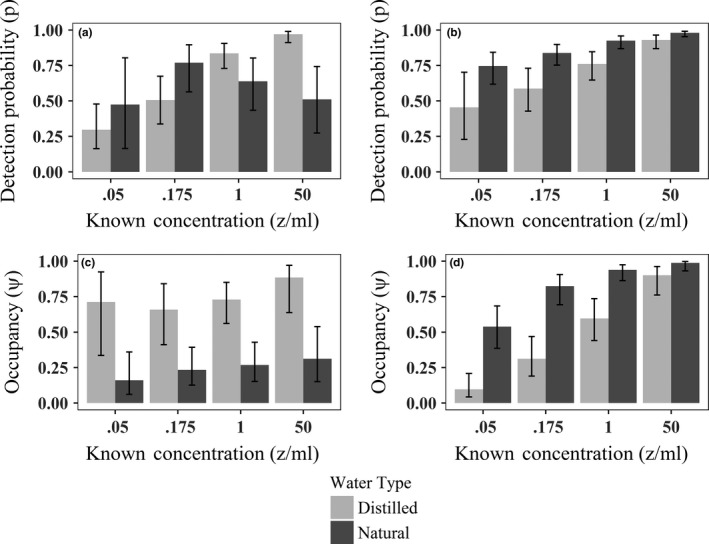
Model‐averaged estimates of *Bd* detection (*p*, a and b) and occupancy probability (*ψ*, c and d) with 95% confidence intervals from standard single sample occupancy analyses when purification methods were (right column) and were not (left column) applied. Occupancy estimates presented here are identical to model‐averaged *Bd* filter occurrence (θ) estimates from the multiscale occupancy analysis

Of the 46 single sample occupancy models fit to the pre‐purification data, only three were supported (Appendix 1A). All supported models included an interactive effect of water type and concentration on detection, and, while detection increased with concentration in distilled water, it was unrelated to concentration in natural water (Figure 3a). The best‐supported covariates for occupancy were an interactive effect of water type and concentration, an additive effect of these factors, and water type alone. Pre‐purification estimates of occurrence in distilled and natural water were biased across concentrations, suggesting that *Bd* occurred in ≤25% of natural water samples and ≤75% of distilled water samples at low concentrations though all jars were inoculated with *Bd* (Figure 3c, Table 1). Post‐purification, a model where both detection and occupancy varied as an additive effect of concentration and water type received 0.85 of the model weight (Appendix 1B). Model‐averaged estimates revealed that post‐purification detection probability for natural water was higher and more closely related to concentration than pre‐purification (Figure 3, top panel). Post‐purification, model‐averaged estimates of *Bd* occurrence increased with concentration in both water types but were still negatively biased at low concentrations (Figure 3d, Table 1). Interestingly, both detection and occupancy probability were estimated to be higher in natural water than in distilled water post‐purification (Figure 3b, d).

When multiple samples were used and purification was not employed, several multiscale occupancy models received support. Best‐supported models generated unbiased estimates of *Bd* occurrence ($\hat{\psi }$ = 1.0) that were constant across treatment types (Table 1, Appendix 2A). However, estimates of *Bd* presence on individual filters (θ) varied with concentration and water type, were biased low, and were identical to *ψ* estimates from the single‐sample occupancy analysis (Figure 3c). The best‐supported models for the post‐purification dataset also suggested a constant occurrence of *Bd* with estimates of 1.0 for all treatment groups (Table 1, Appendix 2B). The estimates of *Bd* availability were once again biased low, but were much higher in natural water than they were pre‐purification (Figure 3d). Detection probability was influenced by concentration and water type in both datasets and was identical to the detection probabilities estimated in the single‐sample occupancy analyses (Figures 3a,b).

### 3.2 Zoospore quantification

We identified five outliers from the pre‐purification dataset and two from the post‐purification dataset. The estimated mean copy number for these outliers was at least an order of magnitude higher than any other sample in the same treatment group and may have resulted from sampling a mass of sporangia dislodged from the culture media. We removed these outliers before assessing the quantification of *Bd* under the different sampling scenarios because we did not think that these samples provided an accurate reflection of zoospore concentrations. Different samples were identified as outliers in the pre‐ vs. post‐purification datasets.

When single samples were considered, the pre‐purification estimate of the correlation between qPCR copy number and known zoospore concentration was positive but low in natural water (*r*_s_ = 0.13, Appendix 3) and was much higher once purification was employed (*r*_s_ = 0.79, Appendix 3). The correlation between qPCR copy number and known *Bd* concentration was high for distilled water when single samples were used, regardless of whether purification had been applied (*r*_s_ = 0.69 pre‐purification versus 0.72 post‐purification, Appendix 3).

**Figure 4:**
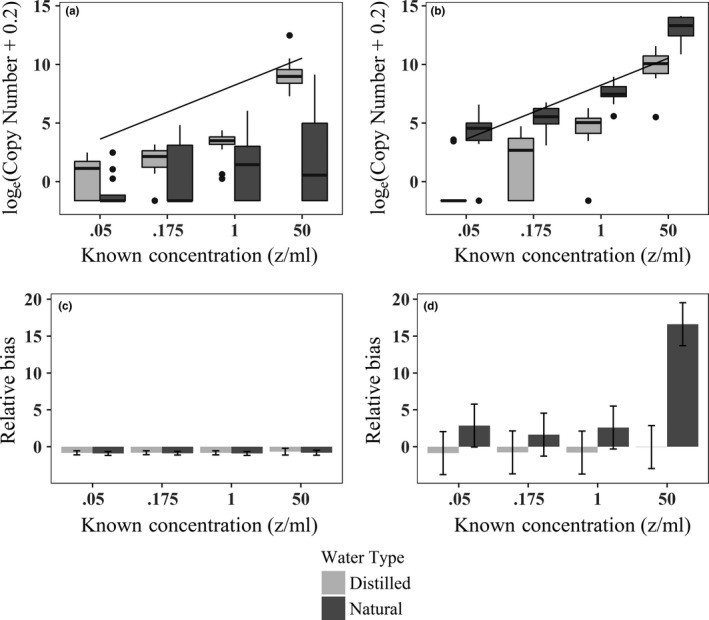
Relationship between qPCR copy number and known experimental concentration (in zoospores/mL) using multiple samples pre‐ (a) and postpurification (b). The line in plots a and b illustrates the 1:1 correspondence between qPCR copy number and known concentration. Model‐averaged estimates and 95% confidence intervals for estimated relative bias in *Bd* concentration from linear regression models before (c) and after (d) purification when multiple samples were used.

Combining multiple samples improved the correlation between qPCR copy number and known zoospore concentration in both water types, but not as much as did the application of the purification protocols in natural water (Figure 4 top panel, Appendix 3). The purification process resulted in a threefold increase in the correlation between qPCR copy number and known zoospore concentration in natural water (*r*_s_ = 0.31 pre‐purification vs *r*_s_ = 0.90 post‐purification, Appendix 3) while no change was seen for distilled water (*r*_s_ = 0.85, Appendix 3) when multiple samples were used.

We found consistent negative bias in estimated pathogen concentration (i.e., the constant model was best‐supported, Appendix 4A) using pre‐purification data (Figure 4c). Post‐purification analyses of relative bias supported an interaction between concentration and water type (Appendix 4B). Zoospore concentration was overestimated in natural water samples post‐purification, especially at the highest concentration, while bias in distilled water remained minimal and negative (Figure 4d). We present relative bias results for only the multiple samples scenario because the findings were identical to those from the single sample scenario.

## 4 DISCUSSION

As the amphibian pathogens *Bd* and *Bsal* become more widespread, amphibian declines are expected to become more common and severe (Yap, Koo, Ambrose, Wake, & Vredenburg, 2015). Researchers and managers need to understand the distribution and dynamics of these pathogens, even at sites where amphibians no longer occur, so that the success of management actions can be assessed. No previous study has experimentally investigated the consequences of imperfect detection and qPCR inhibition on inferences about *Bd* occurrence (Kirshtein et al., 2007; Walker et al., 2007). Further, the only assessment of quantification of *Bd* zoospores using filtration focused on a concentration that was not biologically realistic (>1,000 times higher than our concentrations; Kirshtein et al., 2007). Our work fills these knowledge gaps and yields findings that influence study design, molecular and statistical analyses, and associated biological inferences in amphibian‐pathogen systems or environmental DNA (eDNA) studies.

When multiple samples were collected and qPCR inhibitors were reduced, we reliably detected *Bd* at low concentrations that are likely common in wild systems. A multiscale occupancy approach is a natural fit for pathogen detection data that are imperfect and generated in duplicate or triplicate (Lachish, Gopalaswamy, Knowles, & Sheldon, 2012; McClintock et al., 2010) and such approaches can yield important insights about how covariates influence pathogen distributions. While we are not the first to recommend that conservation biologists and managers should collect multiple samples through time and space to maximize pathogen detection probabilities and to reduce bias in occurrence parameters (Schmidt et al., 2013), we are the first to show how qPCR inhibition and pathogen concentration influence estimates of pathogen occurrence and detection at different scales of interest.

We found that filter‐level *Bd* occurrence (θ) is related to pathogen concentration and suggest exploiting this relationship to explore the factors influencing heterogeneity in *Bd* occurrence across temporal and spatial scales. While we were limited to using site‐level covariates to explore variation in θ, filter‐level covariates including volume filtered, day‐of‐year, position within the wetland, or other factors that may be of biological interest in natural settings. In addition to learning about the processes that influence *Bd* distributions, these insights may help managers identify which candidate reintroduction sites have little or no *Bd,* and subsequently present the lowest risk of disease to reintroduced amphibians. In terrestrial amphibian communities, filtration may be a useful tool for detecting pathogens from fomites or amphibians bathed in water (Hyatt et al., 2007; Shin et al., 2014). In this case, using a framework like the one we present would allow the exploration of site‐ and individual‐level covariates that influence the occurrence (*ψ*) and prevalence (θ) of *Bd* infections.

While three filters were sufficient to obtain unbiased estimates of site‐level occupancy in our experiment, we expect more spatial and temporal heterogeneity in natural settings (Chestnut et al., 2014; Walker et al., 2007) so additional samples will be required, especially at newly invaded sites with low concentrations of *Bd*. Pilot studies where multiple samples are collected and analyzed prior to a larger study can help investigators anticipate plausible detection probabilities in their system and optimize the number of samples needed to address study objectives. Collecting and analyzing multiple samples is expensive, but we have shown that the inferential gains of multiple samples are great. Further, strategies like pooling samples (Hyatt et al., 2007) can reduce laboratory costs.

Previous studies mention a concern for false positives (Olson et al., 2013; Schmidt et al., 2013) and employ thresholds (Shin et al., 2014; Venesky, Liu et al., 2014), indicating that false positives may be more common than is often acknowledged. Precautionary measures including changing gloves frequently, wearing room‐dedicated laboratory coats, cleaning equipment with bleach, and using aerosol‐barrier pipette tips and a dedicated clean room for extraction and qPCR are all best practices that should be incorporated to reduce contamination (Goldberg et al., 2016). In addition, researchers should incorporate negative controls at each level of sample preparation to adequately assess if and when false positives occur (Goldberg et al., 2016). Incorporating an experimentally derived threshold to remove false positives (as we did here) will also remove true detections, resulting in decreased detection probabilities. Statistical methods for estimating this threshold are also available and can be used to differentiate detections from nondetections (Hunter et al., 2017). Finally, model‐based methods to account for false positives also exist, and the best option to account for false positives will likely be context dependent. For instance, if low‐level contamination is seen in the negative controls, qPCR copy number could be used to classify detections as “certain” or “uncertain” (potentially a false positive) using a multiple detection state occupancy model (Miller et al., 2011). The choice of how to appropriately account for false positives is not trivial and has repercussions for estimation of both detection probability and occupancy probability (B.A. Mosher unpublished data, Lahoz‐Monfort, Guillera‐Arroita, & Tingley, 2016).

We caution against using estimates of qPCR copy number as a direct measure of *Bd* abundance without further research, as copy number was biased, especially at high concentrations. Paradoxically, we did find a high correlation between qPCR copy number and concentration treatment group when multiple samples were used, indicating that these copy numbers may still be useful for differentiating between different types of sites that exist in natural settings (e.g., newly invaded [low *Bd* concentration], sublethal/endemic [intermediate *Bd* concentration], and lethal/epidemic [high *Bd* concentration]) using a multistate occupancy approach (MacKenzie, Nichols, Seamans, & Gutiérrez, 2009). Copy number may also be useful for modeling abundance‐induced heterogeneity in detection among occupied sites in the field (Miller et al., 2012), as we found a positive relationship between *Bd* copy number and detection probability when inhibitors were removed. This relationship matched our predictions and reflects that samples with higher *Bd* concentrations are more likely to yield replicates that consistently contain DNA quantities sufficient for detection via qPCR. Our findings mirror those of Clare et al. (2016), who found that while qPCR copy number from swabs could differentiate between moribund and visually healthy individuals, it was not an accurate representation of true infection load. Our study is the first of its kind to investigate how the quantification of *Bd* isolated from the environment is influenced by qPCR inhibition and the collection of multiple samples.

Inhibition of the qPCR strongly influenced *Bd* detection and quantification and can cause negatively biased estimates of *Bd* occurrence and abundance especially when only a single sample is collected. When multiple samples were collected, purifying DNA led to a threefold increase in correlation between copy number and known *Bd* concentration in natural water. We recommend testing for qPCR inhibition in every sample using internal positive controls (IPC) in at least one of the replicate wells (Hyatt et al., 2007). When evidence of qPCR inhibition is found, we recommend that a process to reduce inhibition is used such as the OneStep™ PCR Inhibitor Removal Kit (Zymo Research, Irvine, CA, USA). Other approaches for removing inhibitors should be examined in a rigorous experimental framework similar to what we present here before they are used with field samples. Studies that fail to identify and address qPCR inhibition may grossly underestimate pathogen distributions. Amphibian skin swabs also contain inhibitory agents (Blooi et al., 2013; Kosch & Summers, 2013), and we expect that without purification those sample types are also subject to biased inferences of prevalence (Becker & Zamudio, 2011) or individual infection loads (Stockwell, Garnham, Bower, Clulow, & Mahony, 2016). Surprisingly, we found that inhibitor removal in distilled water resulted in lower (and more biased) *Bd* occurrence estimates (Figure 3c,d), likely due to DNA being lost through the inhibitor removal process (Zymo Research, Irvine, CA). This finding indicates that application of inhibitor removal technology to a sample in the absence of inhibitors may be costly in terms of lost signal and that the optimal protocol may be to analyze samples with and without application of an inhibitor removal step (A.J. Davis unpublished data).

## 5 CONCLUSIONS

Although improving and refining field and laboratory methods for the detection of amphibians and their pathogens is crucial, all sampling methods are imperfect. Thus, “best practices” should include collecting multiple samples, using multiple detection methods, and accounting for imperfect detection using both laboratory and modeling techniques. Employing multiple detection methods on a single site visit creates gains in study efficiency; filter samples for eDNA can complement visual encounter surveys for amphibian detection (Pilliod, Goldberg, Arkle, & Waits, 2013) and filter samples for *Bd* or *Bsal* can complement skin swabs of resident or sentinel individuals for pathogen detection (Schmidt et al., 2013). Performing multiple assays on a single filter sample offers opportunities to detect multiple hosts, pathogens, and other species simultaneously (Blooi et al., 2013).

Existing studies of amphibian‐*Bd* or *Bsal* occurrence dynamics have been limited to studying the prevalence of these pathogens within one or several known host populations (Savage, Sredl, & Zamudio, 2011; Vredenburg et al., 2010). Understanding the dynamics of amphibian hosts and their pathogens at the landscape scale requires the ability to sample each species independently (Mosher et al., 2017) and will yield insights about metapopulation dynamics, long‐term species persistence, and the success of management actions. Our findings are valuable to conservation biologists and managers as they strive to understand and manage complex amphibian‐pathogen systems.

## CONFLICT OF INTEREST

None declared.

## AUTHOR CONTRIBUTIONS

BAM, KPH, and LLB conceived of the experiment. BAM, KPH, LLB, and TC conducted the experiment. TC advised on laboratory protocols. BAM, JDM, and JLK performed the molecular analyses. BAM performed data analyses and wrote the manuscript. All authors edited and revised the manuscript.

## DATA ACCESSIBILITY

Model selection tables and other output: uploaded as online supporting information.

Sample‐specific data are publicly available and can be accessed via the Dryad Digital Repository (https://doi.org/10.5061/dryad.2j0h3).
