# Supplementary material for: Design‐ and model‐based recommendations for detecting and quantifying an amphibian pathogen in environmental samples
Source: Ecol Evol. 2017 Nov 12;7(24):10952–62. doi: 10.1002/ece3.3616 (PMC5743658; doi:10.1002/ece3.3616)
Supplement: Supplementary file 1 [file ECE3-7-10952-s001.docx]

**Appendix 1:** Model selection results for pre-purification (A) and post-purification (B) models applied to *Bd* detection-nondetection data for each inoculated jar (single sample scenario). We examined additive (+) and interactive (*) effects of water type (WT) and concentration (treated as a categorical, Conc.f [factor], or continuous, Conc.c [continuous], variable) for both detection (*p*) and occupancy ($\psi$) parameters, but never included both continuous and factor formulations of concentration in a single model. Here we present only models receiving support, though 46 total models were fit for each analysis. Number of parameters (K), Akaike’s Information Criterion adjusted for small sample sizes (AICc), ΔAICc, model weight, and deviance are given for each model.

| *A. Pre-Purification Single Sample Occupancy* | | | | | |
| --- | --- | --- | --- | --- | --- |
| **Model** | **K** | **AICc** | **ΔAICc** | **Model Weight** | **Deviance** |
| $\psi$(WT*conc.f) $p$(WT*conc.f) | 16 | 685.82 | 0.00 | 0.48 | 651.81 |
| $\psi$(WT+conc.f) $p$(WT*conc.f) | 13 | 686.67 | 0.85 | 0.31 | 659.34 |
| $\psi$(WT) $p$(WT*conc.f) | 10 | 687.53 | 1.71 | 0.20 | 666.73 |
|  |  |  |  |  |  |
| *B. Post-Purification Single Sample Occupancy* | | | | | |
| **Model** | **K** | **AICc** | **ΔAICc** | **Model Weight** | **Deviance** |
| $\psi$(WT+conc.f) $p$(WT+conc.f) | 10 | 644.59 | 0.00 | 0.85 | 623.80 |
| $\psi$(WT+conc.f) $p$(WT*conc.f) | 13 | 649.52 | 4.93 | 0.07 | 622.19 |
| $\psi$(WT*conc.f) $p$(WT+conc.f) | 13 | 649.66 | 5.06 | 0.07 | 622.33 |

**Appendix 2:** Model selection results for multi-scale occupancy models fit to pre-purification (A) and post-purification (B) *Bd* detection-nondetection data using the multiple samples scenario. Model parameters include *Bd* occupancy of the sample unit ($\psi$), *Bd* filter-level occurrence ($\theta$), and detection probability ($p$). We examined additive (+) and interactive (*) effects of concentration (Conc.f [factor] and Conc.c [continuous]) and water type (WT) in both detection, occupancy, and local availability parameters, but never included both continuous and factor formulations of concentration in a single model. Here we present only models receiving support, though 128 total models were fit for each analysis. Number of parameters (K), Akaike’s Information Criterion corrected for small sample size (AICc), ΔAICc, model weights, and deviance are given for each model.

| *A. Pre-Purification Multiple Sample Occupancy* | | | | | |
| --- | --- | --- | --- | --- | --- |
| **Model** | **K** | **AICc** | **ΔAICc** | **Model Weight** | **Deviance** |
| $\psi$(constant)$\theta$(WT) $p$(WT*conc.f) | 11 | 691.87 | 0.00 | 0.37 | 666.73 |
| $\psi$(constant)$\theta$(WT+conc.f) $p$(WT*conc.f) | 14 | 692.53 | 0.65 | 0.27 | 659.34 |
| $\psi$(constant)$\theta$(WT*conc.f) $p$(WT*conc.f) | 17 | 693.65 | 1.78 | 0.15 | 651.81 |
| $\psi$(WT)$\theta$(WT) $p$(WT*conc.f) | 12 | 694.49 | 2.62 | 0.10 | 666.73 |
| $\psi$(WT)$\theta$(WT+conc.f) $p$(WT*conc.f) | 15 | 695.34 | 3.47 | 0.06 | 659.34 |
|  |  |  |  |  |  |
| *B. Post-Purification Multiple Sample Occupancy* | | | | | |
| **Model** | **K** | **AICc** | **ΔAICc** | **Model Weight** | **Deviance** |
| $\psi$(constant)$\theta$(WT+conc.f) $p$(WT+conc.f) | 11.00 | 648.94 | 0.00 | 0.72 | 623.80 |
| $\psi$(WT)$\theta$(WT+conc.f) $p$(WT+conc.f) | 12.00 | 651.56 | 2.62 | 0.19 | 623.80 |

**Appendix 3:** Spearman’s rank-order correlation coefficients (r_s_) for the relationship between qPCR copy number and experimental *Bd* concentration in distilled and natural water samples. Correlation was calculated under the single and multiple samples scenarios, both with and without DNA purification.

|  | **Pre-purification** | |
| --- | --- | --- |
|  | Distilled | Natural |
| Single Sample | 0.69 | 0.13 |
| Multiple Samples | 0.85 | 0.31 |
|  | **Post-purification** | |
|  | Distilled | Natural |
| Single Sample | 0.72 | 0.79 |
| Multiple Samples | 0.85 | 0.90 |

**Appendix 4:** Model selection results for the best-supported linear regression models applied to data on relative bias in estimates of *Bd* concentration from our experiment. We examined additive (+) and interactive (*) effects of inoculated concentration (Conc.f [factor] and water type (WT). Number of parameters (K), Akaike’s Information Criterion for small sample sizes (AICc), ΔAICc, Akaike weights, and deviance are given for each model.

| *A. Pre-Purification Multiple Samples Relative Bias* | | | | | |
| --- | --- | --- | --- | --- | --- |
| **Model** | **K** | **AICc** | **ΔAICc** | **Model Weight** | **Deviance** |
| Constant | 2 | 212.83 | 0.00 | 0.38 | 208.70 |
| WT | 3 | 213.37 | 0.54 | 0.29 | 207.11 |
| Conc.f | 5 | 214.81 | 1.98 | 0.14 | 204.15 |
| WT+Conc.f | 6 | 215.42 | 2.59 | 0.10 | 202.48 |
| WT*Conc.f | 9 | 215.69 | 2.86 | 0.09 | 195.60 |
|  |  |  |  |  |  |
| *B. Post-Purification Multiple Samples Relative Bias* | | | | | |
| **Model** | **K** | **AICc** | **ΔAICc** | **Model Weight** | **Deviance** |
| WT*Conc.f | 9 | 598.74 | 0.00 | 1.00 | 578.65 |
| WT+Conc.f | 6 | 620.60 | 21.87 | 0.00 | 607.66 |
| WT | 3 | 640.72 | 41.99 | 0.00 | 634.46 |
| Conc.f | 5 | 645.36 | 46.62 | 0.00 | 634.70 |
| Constant | 2 | 659.71 | 60.97 | 0.00 | 655.58 |
